# The impact of the COVID-19 pandemic on the mental health of patients diagnosed with inflammatory bowel diseases

**DOI:** 10.25122/jml-2023-0475

**Published:** 2023-12

**Authors:** Oliviu-Florentiu Sarb, Vitalie Vacaras, Vladimir-Petru Filip, Adriana-Daniela Sarb, Roxana-Delia Zaharie, Nicu Draghici, Dafin-Fior Muresanu, Alina-Ioana Tantau

**Affiliations:** 1 Department of Neuroscience, Iuliu Hatieganu University of Medicine and Pharmacy, Cluj-Napoca, Romania; 2 4^th^ Department of Internal Medicine, Iuliu Hatieganu University of Medicine and Pharmacy, Cluj-Napoca, Romania; 3 Department of Infectious Disease, Clinical Hospital of Infectious Disease, Cluj-Napoca, Romania; 4 Heart Institute, Iuliu Hatieganu University of Medicine and Pharmacy, Cluj-Napoca, Romania; 5 Department of Gastroenterology, Octavian Fodor Regional Institute of Gastroenterology and Hepatology, Cluj-Napoca, Cluj, Romania; 6 Department of Gastroenterology, Iuliu Hatieganu University of Medicine and Pharmacy, Cluj-Napoca, Romania

**Keywords:** COVID-19, Crohn’s disease, ulcerative colitis, mental health, depression, stress, anxiety, CD=Crohn's disease, IBD=inflammatory bowel disease(s), UC=ulcerative colitis

## Abstract

Understanding the profound impact of a viral pandemic on the mental health of patients with autoimmune diseases undergoing biological treatment is crucial for future insights. This cross-sectional case-control study aimed to assess the mental health implications of the COVID-19 pandemic on individuals with inflammatory bowel disease (IBD) in Romania, spanning from November 2022 to March 2023. A specialized self-report questionnaire in the Romanian language was developed to measure the multifaceted effects of COVID-19 on the mental well-being of these patients. The findings revealed a significant decline in the mental health of patients with IBD during the pandemic compared to the control group. Patients with IBD exhibited elevated levels of anxiety and concern regarding the virus. Intriguingly, despite the challenges, the vaccination rate was notably higher among patients with IBD, indicating a proactive approach to safeguarding their health. The study also shed light on various coping mechanisms employed by patients with IBD to navigate the pandemic-related restrictions. Engaging in activities such as social media and computer games emerged as effective strategies for managing heightened stress and limitations. In conclusion, the emergence of a novel viral pathogen represents a significant distress factor for patients with autoimmune diseases. Recognizing and comprehending these consequences enhances our understanding of the intricate interplay between physical and mental health and equips authorities with valuable insights to better manage future epidemics or viral outbreaks. This study underscores the importance of tailored support systems and strategies for patients with autoimmune diseases during global health crises.

## INTRODUCTION

The COVID-19 pandemic, affecting over 760 million individuals worldwide between 2020 and 2023, has left an indelible mark, claiming the lives of more than 6.8 million people [1]. The virus, SARS-CoV-2, carries a threat to individuals across all age groups, with heightened risks for those with pre-existing conditions such as cardiovascular disease, chronic kidney disease, chronic respiratory disease, diabetes, neurocognitive disorders, and obesity [2]. Additionally, individuals undergoing immunosuppressive treatments face an elevated risk of complications [2].

The clinical spectrum of COVID-19 spans from asymptomatic cases to critical illness, with varying degrees of severity based on symptoms and physiological markers [2]. In response to the global crisis, the World Health Organization (WHO) issued a pivotal recommendation in December 2020, urging widespread vaccination to curb the spread of the virus and mitigate severe outcomes [3]. The guidance of WHO emphasizes achieving vaccination rates of at least 70% of the population, with priority given to health workers and vulnerable groups, including immunocompromised patients and those with underlying conditions [3].

Patients grappling with inflammatory bowel diseases (IBD), encompassing Crohn’s disease (CD) and ulcerative colitis (UC), face heightened susceptibility to COVID-19 due to immune system dysregulation and the use of immunomodulatory medications [4]. In addition, the pandemic has been associated with an increased incidence of depression, fueled by fears of virus infection and prolonged quarantine periods [5]. Recognizing the broader impact of the pandemic on mental health, fatigue, nutritional status, sleep quality, and the critical choices individuals made regarding vaccination, this study focused on the unique challenges faced by individuals with IBD [6-11]. The absence of a survey in Romanian at the start of this study underscores the novelty and importance of our investigation. This research aimed to serve as a crucial clinical tool, shedding light on the mental health issues precipitated by the COVID-19 pandemic among patients with IBD, offering valuable insights for future healthcare strategies.

## MATERIAL AND METHODS

We conducted an observational cross-sectional case-control study from November 2022 to March 2023 to investigate the impact of the COVID-19 pandemic on the mental health of individuals with IBD. The study focused on patients from medical clinics in Cluj-Napoca, including the Institute of Gastroenterology and Hepatology O. Fodor, the County Emergency Hospital, and the Clinical CF Hospital.

Medical registrars and nurses were provided with anonymous questionnaires, which they then distributed to patients previously diagnosed with IBD during their periodic re-evaluation appointments. Additionally, an online version of the identical questionnaire was shared with individuals who were part of online Facebook support groups for IBD.

Inclusion criteria for the IBD group comprised individuals aged 18 years and above, with a confirmed diagnosis of Crohn's disease (CD) or ulcerative colitis (UC) received before December 2019 and willingness to participate in the study. The control group (CG) consisted of adults aged 18 years or older without an autoimmune disease diagnosis, willing to participate.

The study aimed to collect a minimum of 75 completed questionnaires from participants with IBD and CG. Before data collection, the questionnaires were carefully designed and pre-tested with neurology and psychiatry residents, neurology nurses, and medical school students to ensure their effectiveness. The questionnaire included variables on socio-demographics (age, place of residence - rural or urban, gender - male or female, disease-related history), history of COVID-19 and severity, vaccination status, a comparison of health during the pandemic *versus* the pre-pandemic period, negative feelings experienced during lockdown (rated from 0 to 5), and the effectiveness of coping activities during lockdown (rated from 0 to 10).

Statistical analysis was conducted using IBM SPSS software and Microsoft Excel. For continuous variables, essential statistical measures such as the mean and standard deviation were calculated. Group comparisons were performed using the unpaired t-test for continuous variables and the chi-square test for categorical variables, with a significance level set at 0.05. This rigorous analytical approach ensured a robust examination of the data, facilitating the extraction of meaningful insights and generation of evidence-based conclusions.

## RESULTS

A total of 105 questionnaires were completed by individuals with IBD during the study timeline. To ensure data reliability, we focused our analysis on questionnaires with a completion rate of at least 95%, resulting in the examination of 94 IBD questionnaires (89%). Additionally, a total of 150 questionnaires were completed by the CG, with the statistical analysis conducted on 101 questionnaires (67%).

To establish comparable groups, IBD and CG participants were meticulously matched based on age and gender. Also, matching was done on active smoker status and financial status (Table 1).

**Table 1:** Socio-demographic characteristics of study groups

Variable		IBD group	CG	P value
**Age (years)**		45 (±15.1)	43.2 (±12.7)	0.39
**Gender**	**Male**	48 (51%)	44 (44%)	0.29
	**Female**	46 (49%)	57 (56%)	
**Education (years)**		**14.2 (±3.3)**	12 (±3.1)	**<0.001**
**Active smoker**		26 (28%)	18 (18%)	0.09
**Sleep**	**Hours/day**	6.78 (±1)	**7.1 (±1.01)**	**<0.001**
	**Daytime tiredness**	27 (29%)	22 (22%)	0.26
**Financial status**	**Not enough for a living**	38 (40%)	43 (43%)	0.76
	**No left-over money at the end of the month**	77 (82%)	71 (70%)	0.58
**BMI (Body mass index)**		24.6 (±4.11) kg/m^2^	**27.8 (±5.51) kg/m^2^**	**<0.001**

The mean is displayed for age, education, and sleep (± standard deviation), while absolute numbers (percent) are presented for categorical variables. Variables that were significantly higher are bolded for emphasis.

Upon examination of comorbidities, the control group reported higher incidences of hypertension (32% CG *vs*. 19% IBD), atrial fibrillation (14% CG *vs*. 3% IBD), lung disease (9% CG *vs*. 4% IBD), renal disease (9% CG *vs*. 0% IBD), diabetes mellitus (4% CG *vs* 6% IBD), heart failure (3% CG *vs* 2% IBD), and liver disease (1% CG *vs* 0% IBD) compared to the IBD group. A detailed breakdown of the characteristics of individuals with IBD is presented in Table 2.

**Table 2:** Clinical characteristics and medication used by patients with IBD

	CD	UC	Undetermined colitis
**Number of patients (in total)**	37 (39%)	54 (57%)	3 (3%)
**Number of flares during the last year**	1.7 (±2.78)	1.8 (±1.52)	2 (±0)
**Treatment**			
**Biological therapy**	23 (24%)	35 (37%)	2 (2%)
**Aminosalicylates**	8 (9%)	19 (20%)	1 (1%)
**Immunomodulators**	1 (1%)	0 (0%)	0 (0%)
**Chronic use of corticosteroids**	5 (5%)	0 (0%)	0 (0%)

Data is displayed as absolute numbers (and percent in parentheses) for the number of patients and treatment options and averages (± standard deviation) for the number of flares

The incidence of COVID-19 disease was markedly higher among individuals with IBD (59%) compared to 16% in the CG. Table 3 reveals a significantly elevated risk of COVID-19 disease development in the IBD group. Notably, no critical or severe illness was reported by any respondents. The assessment of disease severity, as detailed in Table 4, demonstrated no significant difference among participants.

**Table 3:** COVID-19 infection rate

	IBD group	CG	RR (95% CI)
**Confirmed COVID-19 infection**	**55 (59%)**	16 (16%)	**3.69 (2.28-5.97)**
**Probable infection (not tested)**	4 (4%)	23 (23%)	0.18 (0.06-0.52)

Data is displayed as absolute numbers (and percent) and relative risk for disease development with its interval

**Table 4:** COVID-19 severity

Disease severity	IBD group	CG	RR (95% CI)
**Moderate illness**	20 (36.3%)	2 (12.5%)	2.9 (0.75-11.1)
**Mild disease**	35 (63.7%)	14 (87.5%)	0.72 (0.55-0.95)

Data is displayed as absolute number (and percent)

Regarding the assessment of vaccination status (Table 5), 73% of patients with IBD received at least one dose of a vaccine, a significant increase from the 48% vaccination rate seen in the CG. However, despite the higher initial vaccination rate, patients with IBD did not receive a significantly greater number of additional shots compared to vaccinated participants in the CG.

Weight gain was reported by 22 patients with IBD (23%), with an average gain of 7.22 kg. In comparison, 12 patients (12%) experienced weight gain in the CG but with a notably higher average gain of 19.8 kg. Conversely, a decrease in weight was observed in 17 patients with IBD (18%), with a mean decrease of 4.6 kg, while in the CG, only seven patients (7%) reported an average mean decrease of 3 kg.

**Table 5:** COVID-19 vaccination assessment

	IBD group	CG	P value
**Vaccination rate**	69 (73%)	48 (48%)	<0.001
**Number of shots**	2.23 (±0.77)	2.17 (±0.78)	

Data is displayed as absolute numbers (and percent) for vaccination rate and mean (± standard deviation) for the number of shots

**Figure 1:**
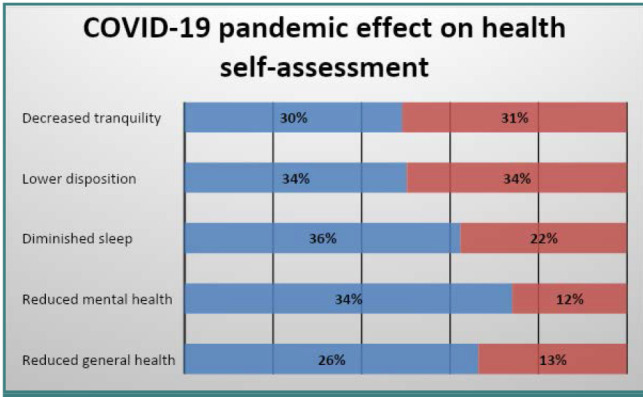
COVID-19 pandemic effect on health self-assessment Blue - IBD group, Red – CG

Participants were asked to identify their greatest fear during the pandemic period. The IBD group predominantly considered the virus the greatest threat, whereas the CG expressed concerns about restrictions first, followed by fears related to the virus. Panic was reported less frequently in both groups. When asked about the impact of the pandemic on their general health, individuals with IBD reported a perceived decline in both mental and overall health, as well as a reduction in sleep quality (Figure 1).

Upon analyzing the impact of negative feelings, we observed significantly higher rates of anger, anxiety, lack of motivation, and domestic and work stress in the IBD group compared to the CG (Table 6).

**Table 6:** Impact of negative feelings

Negative feelings (rated from 0-5)	IBD	CG	P value
**Angerness**	**2.52 (±1.58)**	1.35 (±1.47)	**<0.001**
**Anxiety**	**2.57 (±1.74)**	1.7 (±1.68)	**<0.001**
**Lack of motivation**	**2.16 (±1.73)**	1.33 (±1.53)	**<0.001**
**Domestic stress**	**2.06 (±1.78)**	1.45 (±1.77)	**0.015**
**Workplace stress**	**1.76 (±1.58)**	1.24 (±1.53)	**0.021**
**Boredom**	1.01 (±1.40)	1.2 (±1.50)	0.37
**Suicide intention**	0.8 (±1.47)	0.16 (±0.58)	
**Panic**	1.21 (±1.68)	0.34 (±0.8)	
**Insomnia**	0.86 (±1.28)	0.65 (±1.18)	0.23
**Conspiracy feeling**	1.19 (±1.76)	0.64 (±1.29)	

Data is displayed as mean (± standard deviation)

Respondents were asked about their activities to navigate the challenges of lockdown periods (Table 7). In response, individuals with IBD reported a significantly higher reliance on computer games, internet use, social media, and studying when compared to the CG. Conversely, the IBD group exhibited significantly less engagement in praying and religious interests than the CG.

**Table 7:** Coping activities during the lockdown periods

Coping mechanism	IBD	CG	P value
**Family**	7.2128 (±3.55)	6.2 (±4.3)	0.07
**Physical activity**	5.1596 (±3.92)	5.8 (±3.96)	0.24
**Computer games**	**3.117 (±2.95)**	1.9 (±3.28)	**<0.001**
**Internet, social media**	**4.4681 (±3.63)**	2.2 (±3.14)	**<0.001**
**Pets**	3.117 (±3.78)	3.6 (3.97)	0.57
**Sexual activity**	2.4574 (±3.52)	1.5 (±2.52)	
**Medication**	2.5957 (±3.47)	1.1 (±2.12)	
**Study**	**4.383 (±3.72)**	3.1 (±3.36)	**0.01**
**Smoking**	1.2979 (±2.48)	0.7 (±1.89)	0.06
**Alcohol**	0.734 (±1.6)	0.5 (±1.15)	0.23
**Abuse substances**	0.1383 (±0.7)	0 (±0)	
**Betting**	**0.1383 (±0.7)**	0 (±0)	
**Donations**	2.8085 (±3.14)	1.2 (±2.1)	
**Prayer**	3.1064 (±2.75)	5.7 (±3.94)	**<0.001**
**Work**	5.7128 (±3.09)	6 (±3.89)	0.62

Data is displayed as mean (± standard deviation)

## DISCUSSION

The pre-study phase involved developing the questionnaire based on Romanian standardized questionnaires such as the DASS-21 and the WHO Quality of Life questionnaire [6, 7]. The finalization of the questionnaire involved a two-step process, initially comprising the completion of 15 questionnaires followed by language, grammar, and fluency checks. Subsequently, another round involving 30 questionnaires was conducted, culminating in a final analysis. Feedback from the initial 45 subjects was essential, although it was not incorporated into the final analysis. The study aimed to collect more than 75 questionnaires from each group, a target successfully achieved. However, in the control group, suboptimal questionnaire completion was observed, potentially indicating reduced interest among the subjects. This variability in the quality of the questionnaire emphasizes the importance of considering participant engagement in the subsequent analysis and conclusion.

An intriguing finding was related to the sleep quality of patients with IBD, who reported significantly lower sleep times than the CG. Although increased daytime tiredness was reported more frequently in patients with IBD, this result did not reach statistical significance. This aligns with several other studies that indicated poorer sleep quality in individuals with IBD [9, 10].

A concerning finding from our survey was that nearly half of the participants reported a lack of necessary funds, and almost two-thirds indicated they had no leftover money at the end of the month. This financial strain was observed during a time marked by inflation and the onset of war in regions close to the country, factors that could have significantly influenced these results.

Furthermore, the IBD group displayed a significantly lower mean BMI compared to the CG, likely attributable to clinical manifestations of the disease, such as diarrhea and bloody stools, as well as dietary restrictions imposed by their medical regimen. It is known that individuals with IBD often have a lower BMI [11].

Fear patterns during the pandemic varied between groups, with the IBD group predominantly fearing the virus itself and the severity of the disease, while the CG expressed greater fear of imposed restrictions rather than the virus. This discrepancy may be linked to the heightened awareness of immune system dysregulation and the use of immune modulator treatments in the IBD group, rendering them more susceptible to infections. This awareness likely contributed to a higher vaccination rate in the IBD group, surpassing the recommended 70% threshold set by WHO and other authors [3, 8].

Remarkably, patients with IBD did not express concerns about restrictions, viewing them as useful in reducing the contamination rate. Weight gain during the pandemic emerged as a concern for 23% of patients with IBD, although the mean BMI remained lower than that of the CG.

The coping mechanisms adopted during the pandemic were reflected in the reported activities. Patients with IBD often turned to computer games, internet use, and social media, likely as a reaction to the quarantine restrictions that limited them to their homes. Engaging in studying emerged as a more helpful coping strategy for patients with IBD, suggesting a proactive approach possibly influenced by a higher education level observed in this group. In contrast, prayer was a less common coping strategy among patients with IBD compared to the control group, indicating a reduced reliance on spiritual practices for coping. Despite our findings, other studies showed that the mental health of patients with IBD was negatively affected even before the emergence of the COVID-19 pandemic [12, 13].

This study, meticulously designed to investigate the impact of the COVID-19 pandemic on individuals with IBD, demonstrated a robust methodology, ethical adherence validated by the ethics committee, and reliable data collection and analysis. The findings aligned with existing literature and significantly contributed to our understanding of the nuanced effects of the pandemic on patients with IBD. However, the study faced challenges with participant enrollment due to patient reticence and encountered issues with incomplete questionnaires and participant queries, suggesting potential limitations in engagement. Recognizing these constraints, it is recommended that the questionnaire undergo testing in other studies to enhance its reliability and address any concerns, ensuring a more comprehensive understanding of the study's outcomes. Questionnaires are indispensable tools for comprehensively assessing the intricate impact of infectious diseases on the general population.

## CONCLUSION

Our study, centered on patients with IBD during the COVID-19 pandemic, underscores the importance of creating tailored questionnaires in the absence of standardized tools. The iterative development process, enriched by subject feedback, ensures the reliability and relevance of the information gathered. Noteworthy findings from our study reveal that patients with IBD reported a higher incidence of COVID-19 infection and a greater vaccination rate, although there was no significant risk of severe illness. The pandemic and associated quarantine measures significantly impacted the mental health of patients with IBD, as evidenced by their reports of decreased mental and general health, heightened anger, anxiety, lack of motivation, and increased stress.

Distinct coping mechanisms emerged among individuals with IBD, revealing significant disparities compared to the control group. Patients with IBD relied on computer games, the internet, and social media, engaged in studying, and avoided prayer in significantly different ways compared to the control group. These nuanced insights derived from tailored questionnaires contribute to our understanding of the immediate impact on patients with IBD and provide a valuable framework for future research and interventions.

## Data Availability

Further data is available from the corresponding author upon reasonable request. The online version of the questionnaire is available upon request, as the link was removed when the study was finalized. It may be used freely by any researcher who actively desires after writing a request to the corresponding author.

## Personal thanks

We would like to thank all study participants for taking the time to complete the questionnaires and aid the continuous medical research. We would also like to thank all medical registrators, nurses, and doctors who agreed to help with the study. We would also like to acknowledge the support from our clinic psychologist, Mihaela Prunar, and psychiatrist, Rus Sebastian.
